# Lysine 300 is essential for stability but not for electrogenic transport of the *Escherichia coli* NhaA Na^+^/H^+^ antiporter

**DOI:** 10.1074/jbc.M117.778175

**Published:** 2017-03-22

**Authors:** Octavian Călinescu, Manish Dwivedi, Miyer Patiño-Ruiz, Etana Padan, Klaus Fendler

**Affiliations:** From the ‡ Max-Planck Institute of Biophysics, 60438 Frankfurt am Main, Germany,; the § “Carol Davila” University of Medicine and Pharmacy, 050474 Bucharest, Romania, and; the ¶ Institute of Life Sciences, Hebrew University of Jerusalem, 91904 Jerusalem, Israel

**Keywords:** electrophysiology, enzyme mechanism, membrane transport, site-directed mutagenesis, sodium-proton exchange, transporter, secondary active transport, transport mechanism

## Abstract

Na^+^/H^+^ antiporters are located in the cytoplasmic and intracellular membranes and play crucial roles in regulating intracellular pH, Na^+^, and volume. The NhaA antiporter of *Escherichia coli* is the best studied member of the Na^+^/H^+^ exchanger family and a model system for all related Na^+^/H^+^ exchangers, including eukaryotic representatives. Several amino acid residues are important for the transport activity of NhaA, including Lys-300, a residue that has recently been proposed to carry one of the two H^+^ ions that NhaA exchanges for one Na^+^ ion during one transport cycle. Here, we sought to characterize the effects of mutating Lys-300 of NhaA to amino acid residues containing side chains of different polarity and length (*i.e.* Ala, Arg, Cys, His, Glu, and Leu) on transporter stability and function. Salt resistance assays, acridine-orange fluorescence dequenching, solid supported membrane-based electrophysiology, and differential scanning fluorometry were used to characterize Na^+^ and H^+^ transport, charge translocation, and thermal stability of the different variants. These studies revealed that NhaA could still perform electrogenic Na^+^/H^+^ exchange even in the absence of a protonatable residue at the Lys-300 position. However, all mutants displayed lower thermal stability and reduced ion transport activity compared with the wild-type enzyme, indicating the critical importance of Lys-300 for optimal NhaA structural stability and function. On the basis of these experimental data, we propose a tentative mechanism integrating the functional and structural role of Lys-300.

## Introduction

Living cells are critically dependent on processes that regulate intracellular pH, Na^+^, and volume. Na^+^/H^+^ antiporters, playing a primary role in these homeostatic processes, are located in the cytoplasmic and intracellular membranes of cells (1, 2). Certain human Na^+^/H^+^ antiporters have long been drug targets (3) because they are involved in cardiac failures and other disorders (4). Homologues of EcNhaA^4^ (herein, NhaA), the main *Escherichia coli* Na^+^, Li^+^/H^+^ antiporter, have recently been implicated in the virulence of pathogenic bacteria (5, 6) and in human essential hypertension (7) as well as diabetes (8).

NhaA is characterized by exceptionally high transport activity (9), a stoichiometry of 2H^+^/Na^+^ (10), and a strong pH dependence (9), a property shared with other prokaryotic (11) and eukaryotic Na^+^/H^+^ antiporters (12–15). It is a dimer (16–18), but its functional unit is the monomer (19).

**Figure 1.**
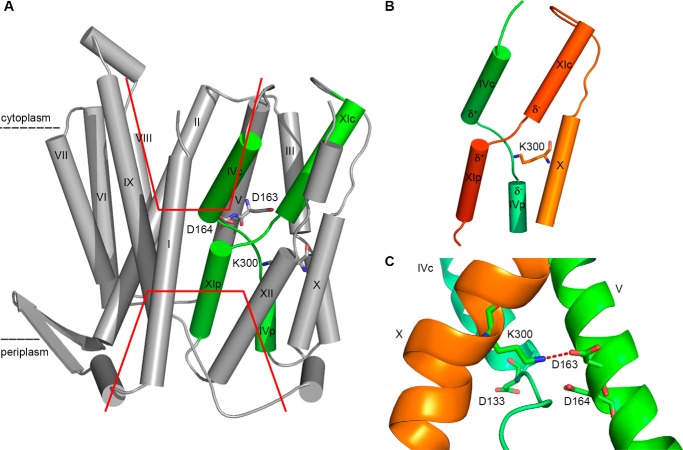
**NhaA structure.** *A*, crystal structure of NhaA at pH 4 (PDB entry 1ZCD), as reported in Hunte *et al.* (20). Roman numerals denote transmembrane helices. Helices IV and XI (in *green*) are interrupted by an unwound portion and are thus split into a cytoplasmic (*c*) and periplasmic (*p*) segment. *Red lines* indicate the cytoplasmic and periplasmic funnels, respectively. *B*, Lys-300 compensates for the partial negative charges (δ^−^) of TMs IVp and XIc of the NhaA structure. The same structure as *panel A* is shown, with TMs other than IV, X, and XI removed for clarity. *C*, the salt bridge (*dashed line*) formed by Lys-300 and Asp-163 of NhaA, as seen in the structure (PDB entry 4AU5) reported in Lee *et al.* (24). The perspective is rotated by ∼180° on the *y* axis relative to *panels A* and *B*.

The crystal structure of the NhaA monomer at acidic pH 4 (20) (Fig. 1*A*) shows that the protein is made up of 12 transmembrane helices (TMs). Six of these TMs form a highly conserved core domain composed of two structurally related helix bundles (TMs III, IV, and V and TMs X, XI, and XII) that are topologically inverted with respect to each other (Fig. 1*A*) (20). TMs IV and XI are each interrupted by an unwound chain that crosses the other chain in the middle of the membrane, leaving two short helices oriented either toward the cytoplasm (c) or toward the periplasm (p) (IVc, IVp, and XIc, XIp, respectively (Fig. 1*A*) (20). The partial positive dipoles of the N termini and the partial negative dipoles of the C termini of the short helices face each other and were suggested to be electrically compensated by Lys-300 and Asp-133, respectively (Fig. 1*B*) (20). This non-canonical TM assembly, termed the NhaA fold (21), is a unique fold that creates a delicately balanced electrostatic environment in the middle of the membrane at the ion-binding site (20, 22). The number of secondary transporters known to share the NhaA fold is steadily increasing (23–28).

Recently, a new structure of NhaA has been determined also at acidic pH 3.5 (24) which showed the NhaA dimer with monomers very similar to those already known (20). It revealed the details of the dimer interface and that TM X is located one helix turn toward the cytoplasm with respect to its location in the original structure. Furthermore, Lys-300 forms a salt bridge with one (Asp-163) of the two aspartate residues (Asp-163, Asp-164) of the cation binding site (Fig. 1*C*). These aspartates are the most evolutionary conserved and absolutely essential residues (29–31). It is still debatable whether the reason for the difference between the two structures is a wrong helix assignment in the original structure or that the two structures represent different conformations. In any event it led to an interesting proposal for a functional role of Lys-300 (24) in the antiporter mechanism.

Being a secondary transporter, the antiport mechanism of NhaA is the canonical alternate accessibility mechanism (32–34). In this mechanism the active site is alternatively exposed to either the cytoplasmic or periplasmic side of the membrane, binding 2H^+^ at one side of the membrane and exchanging them with either Na^+^ or Li^+^ on the other side. In addition to Asp-164, Lys-300 has been suggested to be a proton donor that donates one of the 2H^+^ exchanged (Ref. 24 and see “Discussion”).

However, previous results raised doubts as to the functional role of Lys-300. Lys-300 is not an absolutely irreplaceable residue, a property expected for a proton donor unless other residues can take its role; although mutant K300C did not allow growth of EP432 (an *E. coli* strain deleted of NhaA and NhaB) (35) under extreme stress conditions (0.6 m NaCl at pH 7 or pH 8.3 or 0.2 m LiCl at pH 7) and did not show any Na^+^/H^+^ antiporter activity, it retained 44% Li^+^/H^+^ antiport activity as compared with the WT with ∼200-fold higher apparent *K_m_* (4 mm) (Table 1) (36). Furthermore, the K300E mutation could be genetically suppressed both in *E. coli* and *Helicobacter pylori* NhaA, a close homologue of EcNhaA (37), in the former by mutations in TM IV (36) and TM II and in the latter by mutation in TMII (30, 38).

**Table 1:** **Growth, membrane phenotype and activity results determined by fluorescence dequenching for NhaA Lys300 variants** For characterization of the mutants, *E. coli* EP432 cells transformed with plasmids carrying the indicated mutations were used. Growth experiments were conducted on agar plates with high Na^+^ (0.6 m) or high Li^+^ (0.1 m) at the pH values indicated. +++, the number and size of the colonies after 24 h of incubation at 37 °C was identical to that of the wild type; +, the number of colonies was much lower than that of the wild type; −, no growth. Na^+^/H^+^ and Li^+^/H^+^ antiport activities at pH 8.5 were determined with 10 mm NaCl or LiCl. The activity (maximal level of dequenching) is expressed as the percentage of the positive control, EP432/pAXH3. EP432/pBR322 served as a negative control. The experiments were repeated three times with essentially identical results. ND = not determined.

Mutation	Expression	Growth			Activity		Apparent *K_m_*	
		0.6 m Na^+^		0.1 m Li^+^	Na^+^	Li^+^	Na^+^	Li^+^
		pH 7.0	pH 8.3	pH 7.0				
	%				%		*mm*	
WT	100	+++	+++	+++	100	100	0.5	0.02
K300R*^a^*	60	+++	–	+++	36	93	21.8	0.8
K300H*^a^*	64	+++	+	+++	52	88	7.5	0.24
K300C*^b^*	50	−	−	−	0	44	ND	4
K300A	75	+	−	−	0	30	ND	1.3
K300L	18	−	−	−	0	ND	ND	ND
K300E*^b^*	15	−	−	−	0	0	ND	ND

*^a^* Data are taken from Maes *et al.* (22).

*^b^* Data are from Kozachkov *et al.* (36).

In this study our aim was to investigate the role of Lys-300 in the NhaA antiport mechanism. Does it only have a structural role as suggested by Hunte *et al.* (20), is it primarily relevant for the NhaA transport mechanism as put forward by (24), or possibly both? We, therefore, constructed a number of NhaA variants where Lys-300 was replaced by amino acid residues having a basic (Arg and His), non-polar (Ala, Leu), polar (Cys), or acidic (Glu) side chain and tested the thermal stability and transport activity of these mutants. Based on these experimental results, conclusions are drawn about the role of Lys-300 in the structural and functional properties of the *E. coli* NhaA Na^+^/H^+^ exchanger.

## Results

### Effect of Lys-300 mutations on E. coli salt resistance

To investigate the role of Lys-300 in NhaA functionality, we used previously isolated mutants K300C, K300E (36), K300H, and K300R (22) and constructed K300L and K300A. To characterize the mutants with respect to expression, growth, and antiporter activity in everted isolated membrane vesicles, the mutant plasmids were transformed into EP432, an *E. coli* strain that lacks the two specific Na^+^/H^+^ antiporters NhaA and NhaB (35). This strain neither grows on selective media (0.6 m NaCl at pH 7/pH 8.3 or 0.1 m LiCl at pH 7.0) nor does it exhibit any Na^+^/H^+^ antiporter activity in isolated everted membrane vesicles unless transformed with a plasmid encoding an active antiporter. Whereas variants K300R, K300H, K300A, and K300C were substantially (≥50%) expressed (Table 1) as compared with the level of expression of the WT (100%), mutants K300E and K300L were expressed to 15 and 18% of the control level, respectively (Table 1). However, because all variants are expressed from multicopy plasmids, even a low level of expression is way above the level expressed from a single chromosomal gene, which confers a Na^+^ resistance phenotype (39). Variants K300H and K300R grew similarly to the WT on the selective agar plates at pH 7 but grew slowly (K300H) or did not grow (K300R) on the Na^+^-selective medium at pH 8.3 (Table 1). K300C and K300L did not grow on the selective media, whereas K300A grew with very small colonies only on high Na^+^ at pH 7.

### Antiport activity of Lys-300 mutants in isolated membrane vesicles

**Figure 2.**
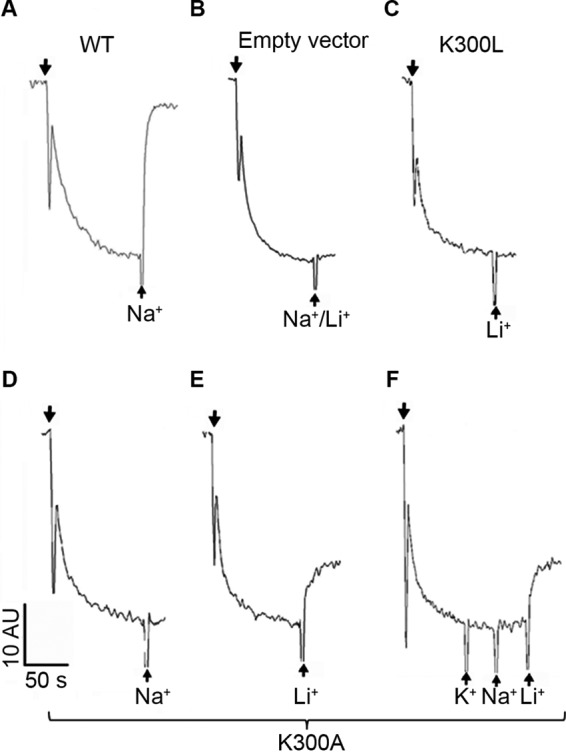
**Antiport activity of NhaA variants K300L and K300A assayed by acridine orange fluorescence dequenching in isolated everted membrane vesicles.** Antiport activities of the membrane vesicles (100 μg of total membrane protein) in a 2.5-ml reaction mixture containing WT NhaA (0.12 μg/ml) (*A*), empty vector (*B*), K300L (0.048 μg/ml) (*C*), or K300A (0.084 μg/ml) (*D–F*). The addition of Tris-d-lactate is indicated by *downward facing arrows*, whereas the addition of 10 mm NaCl or LiCl or KCl is indicated by *upward facing arrows*. Fluorescence is expressed in arbitrary units (*AU*). The experiments were repeated at least three times with practically identical results.

Next, everted membrane vesicles were isolated from each mutant-expressing strain, and the Na^+^/H^+^ antiporter activity was determined. Upon energization by the addition of Tris-d-lactate, the membranes maintained a ΔpH, acidic inside, and the change of ΔpH caused by Na^+^ or Li^+^ addition was monitored using acridine orange as a probe of ΔpH. Although K300L (Fig. 2) and K300E (36) (Table 1) were inactive, mutants K300R and K300H showed substantial Na^+^/Li^+^ antiporter activity (22), and mutants K300C (Table 1) and K300A (Fig. 2) were active only with Li^+^. At saturating cation concentrations, the pH-dependent activity profiles of mutants K300C, K300A, and K300H were very similar to that of the WT (36). In marked contrast, the pH dependence of K300R was shifted to the alkaline side by one pH unit (22).

### Electrophysiological characterization of Lys-300 variants

**Figure 3.**
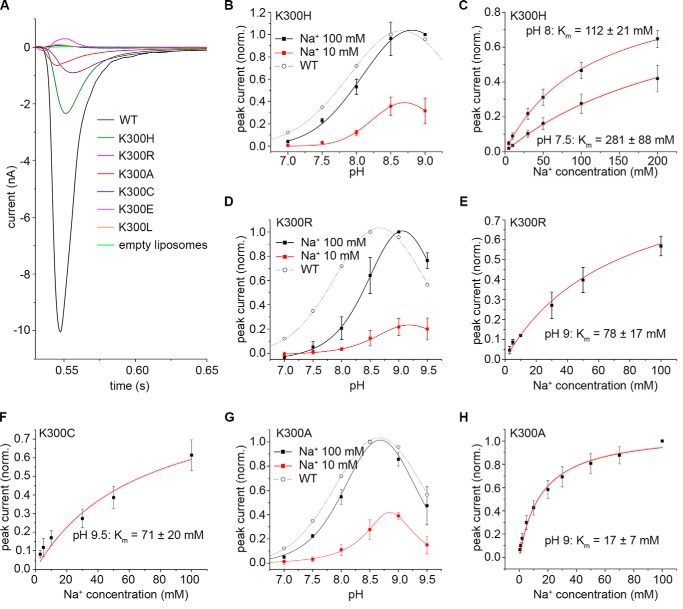
**Electrophysiological analysis of different Lys-300 variants.** *A*, current traces recorded after 100 mm Na^+^ concentration jumps at pH 8.5 for the investigated Lys-300 mutants. The current trace for the WT exchanger was taken from Mager *et al.* (34). *B*, pH dependence of the transient current amplitude recorded for EcNhaA K300H after concentration jumps of 100 mm and 10 mm Na^+^. *C*, Na^+^ dependence of the transient current amplitude recorded for EcNhaA K300H after Na^+^ concentration jumps at pH 7.5 and 8. *D*, pH dependence of the transient current amplitude recorded for EcNhaA K300R after concentration jumps of 100 mm and 10 mm Na^+^. *E*, Na^+^ dependence of the transient current amplitude recorded for EcNhaA K300R after Na^+^ concentration jumps at pH 9. *F*, Na^+^ dependence of the transient current amplitude recorded for EcNhaA K300C after Na^+^ concentration jumps at pH 9.5. *G*, pH dependence of the transient current amplitude recorded for EcNhaA K300A after concentration jumps of 100 mm and 10 mm Na^+^. *H*, Na^+^ dependence of the transient current amplitude recorded for EcNhaA K300A after Na^+^ concentration jumps at pH 9. The WT pH dependence in *B*, *D*, and *G* is given for reference. Curves in *B*, *D*, and *G* are Voigt fits of the experimental data. Curves in *C*, *E*, *F*, and *H* are hyperbolic fits to the data. Data in *B–H* were normalized to the maximum determined amplitude (for *B*, *D*, and *G*) or to the extrapolated maximum of the hyperbolic fits (*C*, *E*, *F*, and *H*). Data in *B–H* are presented as the average of three different recordings ± S.D.

We then tested the electrophysiological behavior of the mutants using solid-supported membrane (SSM)-based electrophysiology. Here, negative currents were observed after Na^+^ concentration jumps at different pH values in mutants K300R, K300H, K300C, and K300A, as for the WT (Fig. 3*A*, Table 2). To decide whether these currents represent steady-state charge transport or a presteady-state charge displacement, it is instructive to compare transient currents from electrogenic WT NhaA (40) and the electroneutral NhaP Na^+^/H^+^ exchangers of *Methanocaldococcus jannaschii* (MjNhaP1) and *Pyrococcus abyssi* (PaNhaP) (41, 42). The former is a prototype of steady-state charge transport, whereas the latter are prototypes of presteady-state charge displacements. WT NhaA transient currents show the following characteristics: 1) They decay slower (∼20 ms; Ref. 40) than presteady-state currents (10 ± 1 ms for MjNhaP1; Ref. 41). 2) Their decay time constants strongly decrease with rising substrate concentration in contrast to those of the Mj and Pa exchangers. 3) Unlike the Mj and Pa exchangers, the currents show alkaline down-regulation, indicating that H^+^ translocation is involved. Overall, the transient currents of the investigated Lys-300 NhaA variants behave like WT NhaA with somewhat modified apparent substrate affinities but with all characteristics 1–3 of WT NhaA; *e.g.* K300A NhaA currents decay with 19 ± 2 ms, their decay times depend on the Na^+^ concentration (supplemental Fig. S1), and their amplitudes are down-regulated at alkaline pH (Fig. 3*G*). Similar conclusions can be drawn for all active Lys-300 NhaA variants that, therefore, represent net steady-state transport of positive charge out of the proteoliposomes (or negative charge into the proteoliposomes) after a Na^+^ concentration jump.

**Table 2:** **Electrophysiological characteristics and thermal stability of Lys-300 variants** Shown is transport activity with Na^+^ as the co-ion. Apparent *K_m_* is taken from the fit of the cation concentration dependence at the given pH. *I*_max_ represents the maximum measured current via SSM-based electrophysiology for the respective transporter (average of 3–4 independent recordings). Thermal stability was assessed by DSF, and melting temperature *T_m_* was determined from the first derivative shown in Fig. 4*B*. ND = not determined.

Mutation	pH_opt_	Apparent *K_m_* (pH)		*I*_max_	Stability
		Na^+^	Li^+^		T*_m_*
		*mm*		*nA*	°*C*
WT*^a^*	8.7	11 ± 1 (8.5)	7.3 (8)	12 ± 2	65.9 ± 0.1
K300R	9.1	78 ± 17 (9)	ND	1.6 ± 0.7	62.4 ± 0.3
K300H	8.8	112 ± 21 (8)	ND	2.2 ± 1	59.9 ± 0.3
K300C	ND	71 ± 20 (9.5)	ND	0.28 ± 0.03	ND
K300A	8.7	17 ± 7 (9)	1.3 ± 0.6 (9)	0.9 ± 0.1	ND
K300L	ND	ND	ND	0	ND
K300E	ND	ND	ND	0	ND

*^a^* Data are taken from Mager *et al.* (34) and from Zuber *et al.* (40).

No transporter-specific currents were observed for K300E and K300L at pH 8.5, the recorded traces for these variants being comparable with those recorded for empty liposomes, devoid of protein (Fig. 3*A*). Furthermore, no transporter-specific currents were recorded for these variants in the entire pH range tested (6.0–9.5). For K300C, whereas no transporter-specific currents were detected at pH 8.5 (Fig. 3*A*), an increase in pH to 9.5 revealed transporter-specific activity (Fig. 3*F*).

A detailed electrophysiological analysis of the active Lys-300 mutants was then performed (Fig. 3, *B–H*). For the K300C mutant, as currents could only be recorded at pH 9.5, the full pH-dependent profile could not be determined. However, we could determine the Na^+^-dependent profile of the currents at pH 9.5, resulting in an apparent Na^+^ affinity of 71 ± 20 mm (Fig. 3*F*). This represents a 10-fold decrease in affinity compared with the value of 7.3 mm determined for the WT at pH 9 (34). Furthermore, the maximum current amplitude of the recorded transients was very low compared with the WT (Table 2). This indicates either reduced transport activity in the mutant (also supported by the activity determined in everted membrane vesicles using acridine orange as a probe of ΔpH across the membrane as shown in Table 1) or that the pH optimum of this mutant lies high in the alkaline range.

In the case of the K300H, K300R, and K300A mutants, a full electrophysiological characterization was possible. All three mutants displayed maximum current amplitudes far reduced compared with the WT protein (Table 2), with maximum currents recorded increasing on average in the order K300A < K300R < K300H. The pH-dependent profile of K300H is highly similar to that of the WT (Fig. 3*B*), with a pH optimum of 8.8. The Na^+^ affinity was also determined for K300H at two different pH values, 8.0 and 7.5 (Fig. 3*C*), and competition between Na^+^ and H^+^ was readily apparent, as is the case for the WT (34).

By comparison, the pH profile of K300R was shifted to the alkaline range compared with the WT or K300H (Fig. 3*D*), with a maximum at pH 9.1. An alkaline shift of the pH dependence of K300R activity was also observed in everted membrane vesicles (22). The determined apparent affinity for Na^+^ (Fig. 3*E*) of K300R was much lower than that determined for the WT (34) at a similar pH (78 mm *versus* 7.3 mm).

K300A is special among the active Lys-300 variants. It has a WT-like pH dependence (Fig. 3*G*) with maximum activity at pH 8.7, but in contrast to other active variants, its apparent Na^+^ affinity is similar to the WT (Table 2). Even more surprising is its extremely high apparent affinity for Li^+^, which agrees with the results of the dequenching assay, where activity was found with Li^+^ but not with Na^+^ (Tables 1 and 2 and Fig. 2). Furthermore, the decay time constants of the Li^+^-induced currents are even larger than those with Na^+^ and depend on the Li^+^ concentration (supplemental Fig. S1). Li^+^/H^+^ exchange of K300A NhaA is, therefore, electrogenic like Na^+^/H^+^ exchange. The same applies to WT NhaA (40).

### Structural stability of investigated NhaA variants

**Figure 4.**
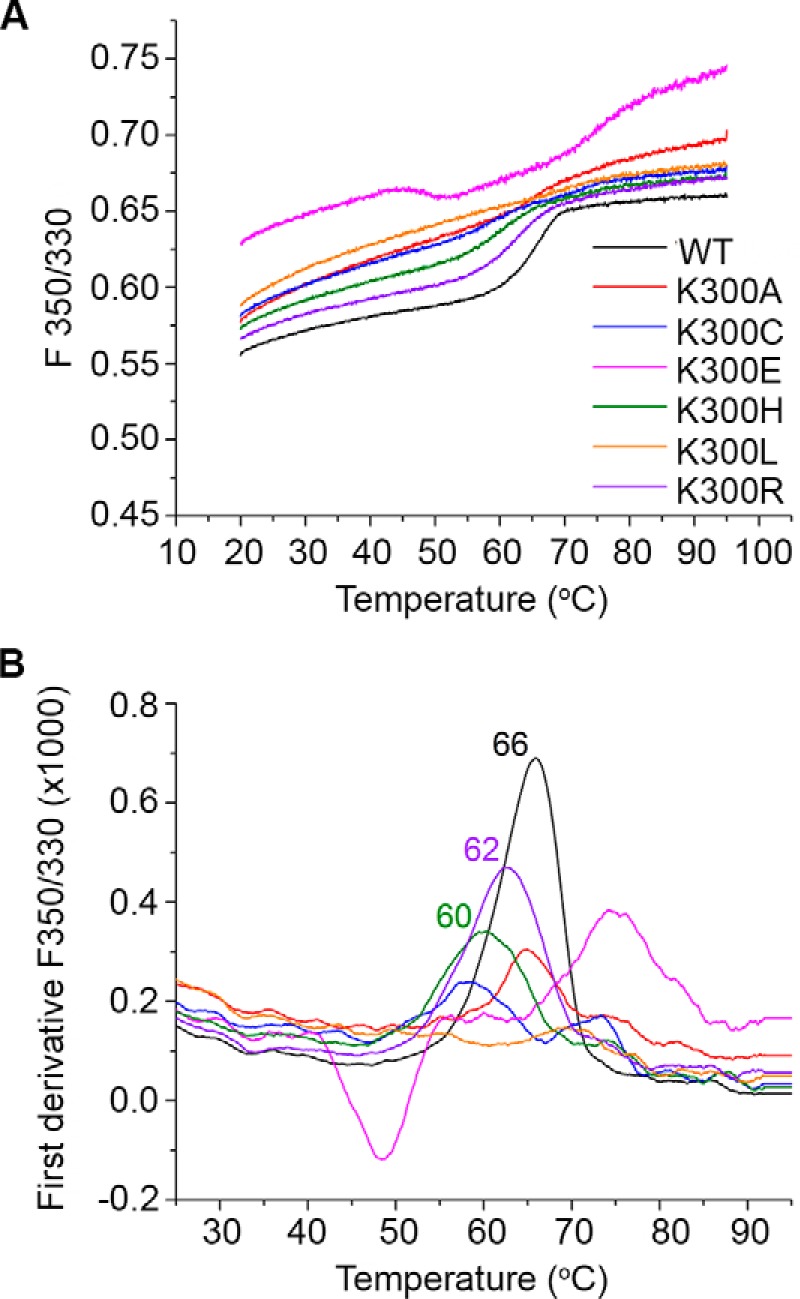
**Thermal stability of the investigated Lys-300 variants.** *A*, melting curves for the EcNhaA protein variants (WT, K300A, K300C, K300E, K300H, K300L, and K300R) at a concentration of 0.5 mg/ml in solution at pH 4. The ratio between fluorescence emission intensities at 350 and 330 nm, respectively (F350/330) was plotted against the temperature, and any pronounced change in this relationship was associated with a protein unfolding event. These traces are representative from three different measurements. *B*, first derivative analysis obtained from the melting curves of the investigated variants. Peak values represent the point at which half of the protein population is unfolded (*T_m_*). Colors of traces in *panel B* are the same as for *panel A*.

In the course of the electrophysiological measurements, it became obvious that only the WT protein produced signals that were stable in magnitude over a long time span (>2 h). For all investigated Lys-300 mutants we could observe high decreases (“run-downs”) in the magnitude of the recorded currents even over relatively short periods of time (10–30 min). These were not reproducible and ranged anywhere from a 10% reduction of the transient current amplitude up to a complete loss of the measured currents. Therefore, we employed differential scanning fluorometry (DSF) in order to quantify the stability of the NhaA Lys-300 mutants and to compare it with that of the WT transporter (Fig. 4). Stability of the NhaA variants was determined by following the fluorescence emission intensity of the protein's tryptophan and tyrosine residues (43) during continuous heating of the protein in solution at pH 4. Thermal unfolding events can be detected thanks to the high sensitivity of these amino acids to changes in their local microenvironment. Every time these residues are exposed to hydrophilic conditions as a consequence of structural changes, their quantum yield decreases, which leads to fluorescence maximum shifts and alterations in fluorescence intensity (43). To account for these two phenomena, the ratio between the emission intensities at 350 nm and 330 nm (F350/330) is plotted against the temperature. The resulting trace is known as melting curve.

A more pronounced inflection point was evidenced in the melting curve for EcNhaA WT in comparison to all Lys-300 mutants (Fig. 4*A*), indicating that any change in this residue compromises thermal stability. The obtained melting temperature for the WT variant was 65.9 ± 0.1 °C, which is close to that reported by Kohlstaedt *et al.* (44) using a thermofluor assay, 66.4 ± 0.6 °C at pH 6.

The extent of the shift in the melting temperature (*T_m_*) for the Lys-300 mutants depended on the side-chain nature. When Lys-300 was exchanged by a residue with a basic side chain (Arg or His), stability was lower, and the transition temperature *T_m_* dropped to 62.4 ± 0.3 °C in K300R and 59.9 ± 0.3 °C in K300H (Fig. 4). Polar (Cys) and nonpolar (Ala, Leu) side chains yielded irregular melting curves with a shallow progression and/or multiple inflection points (multiple maxima in the first derivative), which indicates that a well defined protein melting transition does not take place. The melting curve for the case where a negative residue (Glu) replaced Lys-300 (K300E) presented an anomalous behavior compared with the other investigated variants. Although there were two small inflection points (around 50 and 70 °C, Fig. 4, *A* and *B*), the F350/330 values were higher than those of the other variants, indicating the presence of a considerable population of unfolded protein already at starting temperatures.

Even at room temperature all variants display elevated F350/330 values compared with the WT. Because high F350/330 for this particular protein corresponds to the unfolded state (Fig. 4), this observation points to a reduced structural stability of all variants also at the temperature where the functional assays were performed.

### Functional and structural classification of the investigated Lys-300 variants

Summarizing the structural and functional properties of the investigated Lys-300 variants, we can classify them into three groups, as follows.

#### 

##### WT-like variants (K300H and K300R)

Compared with the WT they have lower but substantial currents, perform well in the growth assay, and display WT-like activity in the biochemical assay. In the DSF assessment, they show a step function characteristic of a well defined protein melting transition and melting temperatures 4 to 6 °C lower than the WT.

##### Variants with low activity (K300C and K300A)

These variants show zero to very small effects in the growth assay, reduced activity in the biochemical assay, and small currents. In the DSF measurement they have no well defined melting transition and display shallow slopes or double peaks in the first derivative.

##### Inactive variants (K300L and K300E)

No activity was detected irrespective of the applied assay. The DSF traces showed no defined melting transition but are characterized by shallow slopes with no peak or inverted and multiple peaks in the first derivative.

## Discussion

Lys-300 plays a central role in the NhaA Na^+^/H^+^ exchanger. Mutation in this position resulted in a certain loss of function in all investigated variants so far. In general, a reduced transport activity may result from impaired functionality but also from structural instability of the protein. Because our electrophysiological analysis revealed indications of protein instability, it was absolutely essential to use an independent method for assessment of protein stability, like the DSF technique, in parallel with the functional characterization.

### Lys-300 stabilizes the NhaA structure

We found a reduced thermal stability of the variants that depended on the character of the amino acid residue in position 300. Only a conservative replacement of Lys-300 by an amino acid with a basic side chain, arginine and histidine, yielded protein with a well defined step function characteristic for a protein melting transition (Fig. 4). The transition temperatures of the variants are 4–6 °C lower than that of the WT, indicating a reduced structural stability. In line with the lower stability of K300H and K300R, a lower activity was detected in the growth, the biochemical, and the electrophysiological assays as compared with the WT.

Low thermal stability and very small or no activity were detected in the other variants, K300C, K300A, K300L, and K300E. In fact, thermal stability and activity seem to be correlated. This indicates that the effect of low activity is at least in part a consequence of the reduced thermal stability rather than directly affecting transport activity. Note that this would not be obvious in an ordinary biochemical assay where the sample is only used once, and a reduced activity would be interpreted as low activity of the particular variant unless special care is taken to detect temporal inactivation of the sample.

The observed structural role of Lys-300 is in agreement with its location in the middle of a most evolutionary-conserved segment (100%) in the middle of TM X (Gly-299, Lys-300, Gly-303) on one side of the helix facing the active site (Fig. 1). Both the original crystal structure of EcNhaA (20) and the more recent one (24) showed that Lys-300 is located between or near the C termini of the short helices IVp and XIc in the middle of the membrane so that it compensates their partial negative dipoles (Fig. 1*B*). The positive charge of Lys-300 has, therefore, been proposed to be important for structural integrity and activity of NhaA (20). Indeed, thermal stability decreases as the nature of the amino acid in position 300 changes from a positively charged (Lys, Arg, and His) to a hydrophobic (Leu, Ala) or negatively charged side chain (Glu).

Alternatively, Lys-300 may be engaged in a salt bridge as proposed by the structure of Lee *et al.* (24). In this case the positive charge of Lys-300 is effectively neutralized by Asp-163 and is unable to compensate the partial negative opposing dipole of the small helices (IVp and XIc). Nevertheless, the salt bridge itself may confer stability to the fold and function as a so-called “ion-lock” as described for the melibiose permease (45).

In conclusion, whether the underlying mechanism be charge compensation or salt-bridge formation, a positive charge in position 300 is indispensable for the structural integrity of NhaA. This has to be taken into account when mechanistic conclusions are drawn based on a comparison of the activities of Lys-300 variants. On the other hand, the mutation clearly affects also the transport mechanism directly as evidenced by the cation affinity drop of most variants. The functional role of Lys-300 will be addressed in the following.

### Lys-300 is not essential for electrogenic Na^+^/H^+^ exchange

NhaA, like most (and possibly all) members of the CPA2 evolutionary branch (CPA = cation proton antiporter) of cation proton antiporters, is electrogenic; that is, it functions with a H^+^/Na^+^, Li^+^ stoichiometry >1 (for NhaA a 2H^+^/1Na^+^ stoichiometry was determined in Refs. 9 and 33). If Lys-300 is an essential proton donor/acceptor in the NhaA reaction mechanism, electrogenicity of Na^+^/H^+^ exchange can only be conserved if it is exchanged for a residue that can bind a H^+^ ion. Indeed, mutants K300H and K300R, where Lys-300 is exchanged for an amino acid with a basic side chain, exhibited substantial electrogenic Na^+^/H^+^ antiporter activity (Fig. 3 and Table 2). Although the apparent *K_m_* values of these variants were 10-fold higher than that of the WT with both substrates (Table 1), they both retained Na^+^/H^+^ antiporter activity in everted membrane vesicles (52 and 36%, respectively) and Li^+^/H^+^ activity (88 and 93%, respectively) at pH 8.5 and grew under several selective growth conditions (Table 1 and Ref. 22).

Surprisingly, the variants K300C and K300A, where the side chain in position 300 most likely cannot protonate, not only exhibited substantial Li^+^/H^+^ antiporter activity in everted membrane vesicles (44 and 30%, respectively, compared with WT, Table 1) but were also electrogenic (Fig. 3 and Table 2). Although the negative electrophysiological signals obtained with K300C and K300A were consistently smaller compared with WT, they clearly indicate a H^+^/Na^+^ transport stoichiometry of >1.

Only K300L and K300E were inactive in the biochemical and electrophysiological assays (Tables 1 and 2). Although Glu-300 in the K300E variant is a potential proton donor/acceptor, no transport activity was detected possibly due to a lack of protein stability suggested by its very irregular melting curve (Fig. 4*A*).

**Figure 5.**
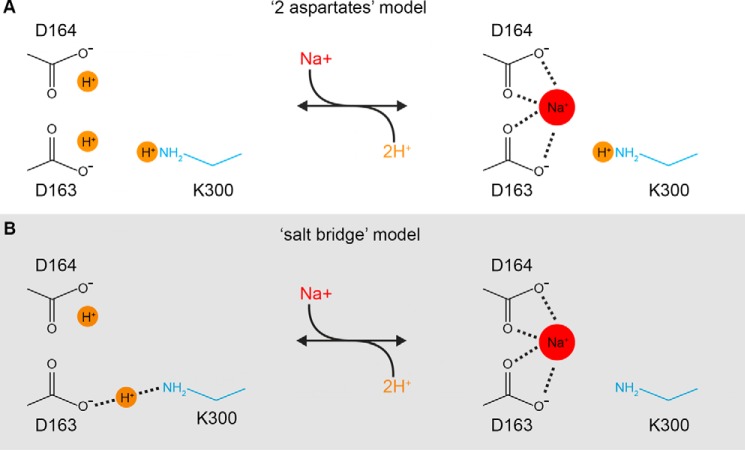
**Mechanistic models for Na^+^/H^+^ exchange in NhaA.** The NhaA transport mechanism consists of two Na^+^/H^+^ exchange reactions separated by conformational transitions changing accessibility of the substrate binding site from periplasmic to cytoplasmic and back. The figure shows these exchange reactions for the two different transport mechanisms proposed for NhaA. *A*, in the two-aspartates model (20), the two transported protons bind to the two aspartic acids. *B*, in the salt-bridge model (24) one proton resides on Asp-164, and the second proton resides in the Asp163–Lys-300 salt bridge. For further discussion of the models see “Discussion.”

As the electrophysiological data demonstrated that no active mutant is electroneutral, there are two possible explanations for our result. Either Lys-300 has no role in the Na^+^/H^+^ exchange mechanism and the lower activity of K300C and K300A is due to structural instability, or Lys-300 is indeed a proton acceptor/donor but in its absence there is an alternative mechanism, namely that the two aspartates Asp-163 and Asp-164 can function as proton acceptors/donors but with lower effectiveness. In this case, the two transport mechanisms discussed below and shown in Fig. 5 are not mutually exclusive.

### Previously suggested models for the NhaA transport mechanism

Similar to the original crystal structure of NhaA (20) the new structure by Lee *et al.* (24) demonstrated the inward facing conformation of the transporter at low pH while additionally revealing that the positively charged amine of Lys-300 forms a salt bridge with Asp-163 (24). This observation led to the suggestion of a “salt-bridge” transport mechanism (24) as shown in Fig. 5, which compares it to the conventional two-aspartates mechanism (20).

As characteristic for a secondary active transporter, the NhaA transport mechanism consists of at least two Na^+^/H^+^ exchange reactions separated by conformational transitions changing accessibility of the substrate binding site from periplasmic to cytoplasmic and back (32, 34). In the conventional two-aspartates model (20) the two transported protons bind to the two aspartic acid residues and are released to the cytoplasm by Na^+^ binding to the aspartates (Fig. 5*A*). In the “salt-bridge model” one proton resides on Asp-164 and the second proton in the Asp-163–Lys-300 salt bridge (Fig. 5*B*). As suggested previously (34), when intracellular pH becomes more alkaline, intracellular Na^+^ ions successfully compete with protons for binding to the proton acceptors in the binding site. In the two-aspartates model this leads to the displacement of the two protons from the two aspartates. In the salt-bridge model upon Na^+^ binding, the first Asp-164 releases its proton. Molecular dynamics simulations (24) showed that subsequently Asp-163 switches from interacting with Lys-300 to contributing to binding the Na^+^ ion, and a second H^+^ is released from Lys-300. The Na^+^-bound form of the transporter would then switch to the outward-facing conformation where the sodium ion can be released, the salt bridge can reform by binding of H^+^ to Lys-300 and to Asp-164, and finally the transporter switches back to the inward-facing conformation.

In summary, in the salt bridge mechanism proposed by Lee *et al.* (24), Lys-300 plays a role of an essential proton donor/acceptor in the NhaA reaction cycle. This implies that replacing Lys-300 of NhaA by a residue that cannot protonate will yield either an inactive mutant or a variant, which is electroneutral, a conclusion that is not in agreement with our experiments.

The arguments given above would obviously be in favor of the two-aspartates model. However, there is a third possibility, namely that the transporter, after replacement of Lys-300 by an uncharged residue like alanine, switches from the salt-bridge model to the “two aspartates” model. The considerable lower currents recorded in this case may be explained by the fact that the transporter is optimized for the salt-bridge model and works with lower turnover in the two-aspartates model. In both cases transport is electrogenic, as experimentally demonstrated in the electrophysiological experiments. An electroneutral mechanism where only one H^+^ is bound and exchanged for one Na^+^ ion can be ruled out.

### Conclusions and a hypothetical transport mechanism

In conclusion, the two major contributions of the present study are the experimental proof that Lys-300 is important for the stability of the transporter and, although not essential, may play an important role for an effective transport process. Electrogenic Na^+^/H^+^ exchange can do without it, although with reduced capacity. It is, however, possible that the functional importance attributed to Lys-300 is at least in part a consequence of its structural role.

Given that Lys-300 stabilizes the structure of the NhaA Na^+^/H^+^ exchanger by engaging in a salt bridge with Asp-163, we can also envision that this is important for the dynamics of the transport process. An effective strictly coupled Na^+^/H^+^ exchange process requires that only the fully loaded transporter (2 H^+^ or 1 Na^+^ bound in the case of NhaA) can perform this transition and that it is inhibited in the empty apo transporter (46). We suggest that the formation of the Lys-300–Asp-163 salt bridge may be an inhibitory element by making the structure more rigid so that the energetic barrier for the conformational transition of the apo transporter is increased. Binding of substrates (Na^+^ or H^+^) would then break the salt bridge and allow the transitions of the loaded transporter.

In this concept the transport process proceeds as follows. 1) In the periplasmic open conformation without substrates Asp-164 is unprotonated, and Asp-163 is engaged in a salt bridge with Lys-300, which stabilizes this conformation. 2) When two H^+^ ions bind from the periplasmic side to Asp-163 and Asp-164, the salt bridge is broken, the structure becomes less rigid, and a conformational transition can take place altering the accessibility and releasing the transported H^+^ ions to the cytoplasmic space. 3) Because now Asp-163 is unprotonated, it can again form a salt bridge with Lys-300, effectively inhibiting the reorientation of the unloaded transporter. 4) When Na^+^ binds from the cytoplasm, the salt bridge is broken as demonstrated recently by a molecular dynamics study (24), and a conformational transition allows Na^+^ release at the periplasmic side of the membrane.

The transport mechanism outline above elegantly explains why Lys-300 apparently has a dual role, structural as well as functional, and is consistent with the data provided by Lee *et al.* (24). If replaced by an uncharged residue like alanine the Na^+^/H^+^ exchanger would still be functional but partly uncoupled, which compromises its function especially when substrate gradients are present. This may explain why K300A NhaA is still functional but with low activity. Experimental efforts to demonstrate the uncoupled function of Lys-300 mutants like K300A NhaA will be required to substantiate this hypothetical mechanism.

Finally, we would like to stress the importance of understanding the transport mechanism of *E. coli* NhaA. Although the prokaryotic NhaA is evolutionarily remote from the eukaryotic sodium/proton exchangers (NHEs and NHAs), we successfully modeled NHE1 and NHA2 on the basis of the crystal structure of NhaA (47, 48) Therefore, the results presented here can guide experiments that would lead to a better understanding of the functionality also of the human antiporters.

## Experimental procedures

### Genetic constructs

Mutant variants of NhaA in which Lys-300 was replaced were obtained in plasmid pAXH3, a pET20b derivative (19). The preparation of mutants K300E and K300C was previously described in Kozachkov *et al.* (36), whereas K300R and K300H were first described in Maes *et al.* (22). The mutants K300L and K300A were obtained by site-directed mutagenesis using a PCR-based protocol with pAXH3 as a template. The *nhaA* gene DNA of each construct was sequenced to verify the mutation.

### Salt resistance assays

Survival of *E. coli* EP432 (35) expressing NhaA variants K300L and K300A under conditions of high concentrations of Na^+^ or Li^+^ was assessed as previously described (22).

### Determination of Na^+^, Li^+^/H^+^ antiporter activity in isolated everted membrane vesicles

Everted membrane vesicles from EP432 transformed with the respective plasmids were prepared as previously described (49). Everted membrane vesicles were used to determine Na^+^/H^+^ or Li^+^/H^+^ antiporter activity with an assay based on the measurement of Na^+^- or Li^+^-induced changes in the ΔpH as measured by acridine orange, a fluorescent probe of ΔpH (50). The fluorescence assay was performed in a 2.5-ml reaction mixture containing 100–150 μg of membrane protein, 0.1 μm acridine orange, 150 mm choline chloride, 50 mm Bis-tris propane, and 5 mm MgCl_2_, and pH was titrated with HCl. Membrane vesicles were acidified by the addition of 2 mm Tris-d-lactate, inducing fluorescence quenching of the acridine orange dye. Dequenching of fluorescence upon the addition of either Na^+^ or Li^+^ indicates that protons are exiting the vesicles in antiport with either cation. As shown previously (51), the end level of dequenching is a good estimate of antiporter activity, and the ion concentration that gives half-maximal dequenching is a good estimate of the apparent *K_m_* of the antiporter activity. For determination of the apparent *K_m_*, the end level of dequenching for different concentrations of the tested cations (0.01–100 mm) at the indicated pH levels was used, and the apparent *K_m_* values were calculated by linear regression of a Lineweaver-Burk plot.

### Overexpression, purification, and reconstitution

C-terminally His-tagged proteins were overexpressed in *E. coli* BL21(DE3) cells and purified using immobilized metal affinity chromatography as previously described (2). Reconstitution of the purified proteins into proteoliposomes was performed using *E. coli* polar lipid extract (Avanti Polar Lipids, Alabaster, AL) at a lipid-to-protein ratio of 5, essentially as previously described (34).

### SSM-based electrophysiology

Electrophysiological measurements were performed essentially as previously described (34). In brief, 30 μl of proteoliposomes at a lipid-to-protein ratio of 5 were added to the SSM sensor and allowed to adsorb for at least 1 h on top of the preformed octadecanethiol/phospholipid hybrid bilayer. Transient currents were also recorded for Na^+^ concentration jumps performed on “empty” liposomes that did not contain transporter proteins.

A single solution exchange protocol (52) was employed, with solutions exchanged over the SSM sensor in the order non-activating–activating–non-activating. All solutions contained 25 mm MES, 25 mm MOPS, 25 mm Tris, 100 mm KCl, 5 mm MgCl_2_, and 1 mm dithiothreitol and were titrated to the desired pH with HCl or KOH. In addition, non-activating solutions contained 200 mm extra KCl, whereas activating solutions contained *x* mm NaCl and (200 − *x*) mm KCl instead. A similar protocol exchanging NaCl for LiCl was employed for measuring Li^+^-dependent transport activity.

The amplitude of the recorded transient currents was used as a measure of steady-state transport activity. Recorded currents were corrected by subtracting the amplitude of transients generated by solution exchange effects recorded on the same sensor in the case where the latter were substantial compared with the transporter-dependent transients.

### Differential scanning fluorometry

Protein stability of the purified NhaA variants (WT and K300*X*, *X* = Ala, Cys, Glu, His, Leu, Arg) was analyzed by DSF. Glass capillaries were loaded with ∼10 μl of protein at a concentration of 0.5 mg/ml in buffer containing 100 mm KCl, 5 mm MgCl_2_, 0.03% DDM, 25 mm potassium acetate (pH 4), and placed in the thermal plate of a Prometheus NT.48 instrument (NanoTemper Technologies, Munich, Germany). Temperature was increased in a range of 20 to 95 °C at a heating rate of 1 °C/min. Thermal protein unfolding was followed by monitoring the tryptophan fluorescence at emission wavelengths of 350 and 330 nm upon excitation at 280 nm with an excitation power setting of 10%.

Melting curves were obtained by plotting the ratio of the two emission intensities (F350/330) *versus* the temperature. The thermal unfolding transition midpoint or melting temperature (*T_m_*, °C) corresponded to the inflection point of the melting curves and was estimated via first derivative analysis.

## Author contributions

E. P. and K. F. initiated and directed the project. O. C., M. D., and M. P.-R. performed the experiments described. O. C., M. D., M. P.-R., E. P., and K. F. analyzed the data and wrote the manuscript. All authors reviewed the results and approved the final version of the manuscript.

## Supplementary Material

Supplemental Data

## Footnotes

4 The abbreviations used are:

EcNhaA: NhaA Na^+^/H^+^ exchanger of *E. coli*
TM: transmembrane segment
DSF: differential scanning fluorometry
F350/330: fluorescence emission ratio at 350 and 330 nm
SSM: solid-supported membrane
Bis-tris propane: 1,3-bis[tris(hydroxymethyl)methylamino]propane.
