# Supplementary material for: Lysine 300 is essential for stability but not for electrogenic transport of the Escherichia coli NhaA Na+/H+ antiporter
Source: J Biol Chem. 2017 Mar 22;292(19):7932–41. doi: 10.1074/jbc.M117.778175 (PMC5427271; doi:10.1074/jbc.M117.778175)
Supplement: Supplemental Data [file supp_292_19_7932__index.html]

Lysine300 Is Essential for Stability but not for Electrogenic Transport of the E. coli NhaA Na+/H+ Antiporter — Lysine 300 is essential for stability but not for electrogenic transport of the Escherichia coli NhaA Na+/H+ antiporter — Lysine 300 is essential for stability of E. coli NhaA — Supplemental Data 

# Lysine 300 is essential for stability but not for electrogenic transport of the *Escherichia coli* NhaA Na+/H+ antiporter

## Supplemental Data

- Supplemental Data (.pdf, 268 KB) - Supplemental Data
