# Supplementary material for: Lysine 300 is essential for stability but not for electrogenic transport of the Escherichia coli NhaA Na+/H+ antiporter
Source: J Biol Chem. 2017 Mar 22;292(19):7932–41. doi: 10.1074/jbc.M117.778175 (PMC5427271; doi:10.1074/jbc.M117.778175)
Supplement: Supplemental Data [file 10.1074_M117.778175_jbc.M117.778175-1.pdf]

Lysine300 is Essential for Stability but not for Electrogenic Transport of the *E. coli* NhaA Na^+/H^+ Antiporter

Octavian Călinescu, Manish Dwivedi, Miyer Patino-Ruiz, Etana Padan, and Klaus Fendler

Supplemental Figure S1

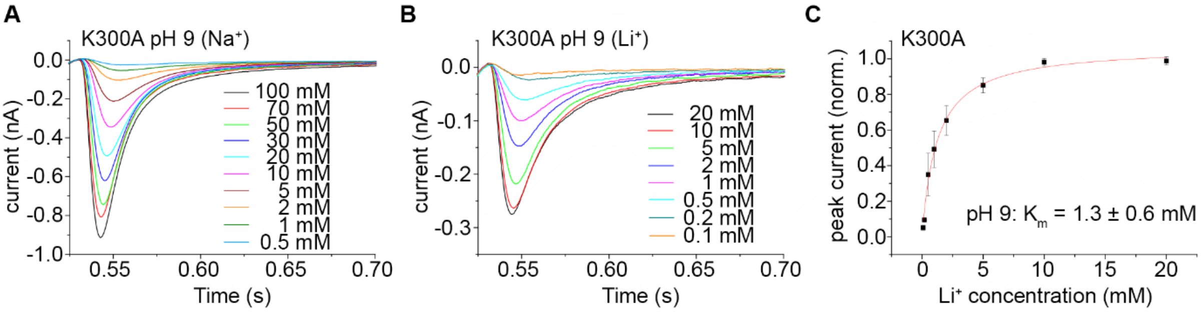

Figure S1. Na^+ and Li^+ concentration dependence of NhaA K300A assessed by SSM-based electrophysiology. A, Current traces generated by different Na^+ concentration jumps in the range 0.5 - 100 mM at pH 9. B, Current traces generated by Li^+ concentration jumps in the range 0.1 - 20 mM at pH 9. C, Li^+ dependence of transient current amplitudes recorded with Li^+ concentration jumps at pH 9. Data in A and B are representative from three replicates. Data in C were normalized to the maximum determined amplitude, fitted to a hyperbolic function and represent the average of three different recordings ± s.d.
